# Advancements in Skeletal Tumour Management: Linking Metastatic Biology With Surgical Reconstruction

**DOI:** 10.7759/cureus.108278

**Published:** 2026-05-05

**Authors:** Eswara Reddy G, Shivakumar MS, Sameer A Ansari, Manjunath A N, K. Parameswaran Namboothiri, Syed F Hamid

**Affiliations:** 1 Department of Orthopaedics, MVJ Medical College and Research Hospital, Bangalore, IND; 2 Department of Orthopaedics, Hamdard Institute of Medical Science and Research, New Delhi, IND; 3 Department of Orthopaedics, Grass Life Hospital, Chennnarayapatna, IND; 4 Department of Panchakarma, Amrita School of Ayurveda, Amrita Vishwa Vidyapeetham, Amritapuri, IND; 5 Department of Nursing, SPHE College of Nursing, Gharuan, IND

**Keywords:** bone metastasis, endoprosthesis, orthopaedic oncology, skeletal tumours, tumour microenvironment

## Abstract

Skeletal tumours, particularly metastatic bone disease, represent a significant clinical challenge due to their complex biological behaviour and impact on structural integrity and patient function, with improved cancer survival contributing to increased skeletal involvement requiring integrated management. The objective of this review is to examine the evolving relationship between metastatic biology and surgical reconstruction, with emphasis on translational insights for clinical decision-making. A structured narrative review was conducted using PubMed, Scopus, and Web of Science to identify literature published between 2015 and 2025, employing predefined search terms including "bone metastasis", "tumour microenvironment", "skeletal tumours", "MRI", "PET/CT", "radiomics", "targeted therapy", "immunotherapy", and "orthopaedic reconstruction". Studies were screened through title and abstract review followed by full-text assessment, with inclusion criteria prioritising peer-reviewed clinical studies, systematic reviews, and translational research relevant to molecular mechanisms, imaging, systemic therapies, and reconstructive strategies; methodological quality and relevance were appraised qualitatively, and findings were synthesised using a thematic integrative framework. Current evidence highlights tumour-bone interactions, including chemokine-mediated homing and RANK/RANKL pathway dysregulation, as key drivers of disease progression, while advances in imaging and radiomics improve diagnostic accuracy and prognostication; comparative analysis indicates that MRI provides superior local tumour delineation, whereas PET/CT enables assessment of metabolic activity and systemic disease burden, thereby informing surgical planning. Systemic therapies influence tumour biology and surgical timing, and innovations in endoprosthetic and biological reconstruction have expanded limb-salvage options. Key outcomes of this synthesis demonstrate that integrating molecular pathways, imaging-derived biomarkers, and treatment response parameters into surgical decision-making enhances prognostic stratification, optimises intervention timing, and improves functional outcomes, although heterogeneity in study design limits standardisation. This review moves beyond descriptive synthesis by providing a clinically oriented integrative framework linking metastatic biology, radiological phenotype, and reconstructive strategy selection, supporting personalised, multidisciplinary care and improved clinical outcomes in orthopaedic oncology.

## Introduction and background

Skeletal tumours represent a heterogeneous group of neoplastic pathologies, comprising primary bone malignancies and secondary metastatic lesions, with the majority of adult cases related to metastatic disease [1]. Advances in the treatment of systemic cancer have led to improved survival across multiple malignancies, resulting in a corresponding increase in skeletal involvement during disease progression [2]. Bone metastases most commonly arise from breast, prostate, lung, and renal cancers, reflecting complex biological interactions between circulating tumour cells and the specialised bone microenvironment [3]. This increasing burden poses significant clinical challenges, including pain, pathological fractures, spinal cord compression, and functional impairment, which collectively contribute to reduced quality of life and increased healthcare utilisation [4].

Skeletal metastasis has a biological basis; it is characterised by dynamic and reciprocal interactions between tumour cells and the bone microenvironment, conceptualised by the seed-and-soil paradigm. Bone-selective homing through chemokine gradients, adhesion molecules, and vascular niches facilitates targeted tumour cell colonisation [5]. Once established, metastatic cells disrupt bone homeostasis by dysregulating signalling pathways such as the receptor activator of nuclear factor kappa-B (RANK)/ receptor activator of nuclear factor kappa-B ligand (RANKL)/ osteoprotegerin axis, leading to osteolytic or osteoblastic lesions depending on tumour phenotype [6]. These processes create a self-perpetuating cycle of bone destruction or aberrant formation, whereby the release of growth factors further amplifies tumour proliferation and local progression. Recent molecular insights have identified additional contributors, including immune modulation, exosomal communication, and metabolic reprogramming, which refer to changes in immune response, cell-to-cell signalling, and tumour energy utilisation, collectively modulating metastatic niche formation and therapeutic responsiveness [7].

Simultaneously, advances in tumour biology have been paralleled by significant improvements in diagnostic and imaging modalities, enabling earlier detection and more precise lesion characterisation [8]. High-resolution magnetic resonance imaging, positron emission tomography, and hybrid imaging platforms provide detailed evaluation of tumour extent, biological activity, and treatment response. Radiomics and artificial intelligence-based analyses extract quantitative features from medical images, enabling improved prognostication and risk stratification. However, comparative integration of these modalities remains limited in routine clinical workflows; MRI offers superior soft tissue and marrow delineation, whereas PET/CT provides critical metabolic and systemic disease assessment, yet their combined interpretation is not consistently operationalised in surgical planning. Despite these technological advancements, the translation of imaging-derived biological insights into surgical planning remains inconsistent, with limited standardisation in how radiological biomarkers influence operative decision-making, implant selection, and risk stratification. From a radiological standpoint, imaging should function not only as a diagnostic modality but also as a decision-enabling tool that stratifies surgical risk, predicts structural failure, and guides reconstruction strategy. However, current clinical practice lacks standardised frameworks that translate imaging-derived biomarkers into operative decision pathways. This limitation restricts the use of imaging for prognostic modelling and personalised surgical planning, representing a critical unmet need in orthopaedic oncology. This lack of integration represents a key translational gap between diagnostic capability and surgical execution in orthopaedic oncology practice.

Surgery remains a cornerstone in the management of skeletal tumours, encompassing both curative approaches for selected primary malignancies and palliative interventions aimed at restoring function and stability in metastatic disease [5]. Over recent decades, there has been a paradigm shift from radical ablative procedures to limb-salvage techniques, supported by advances in endoprosthetic design, biomaterials, and reconstructive strategies [3]. Modular prostheses, improved fixation techniques, and patient-specific implants have expanded reconstructive possibilities, even in anatomically complex regions. Concurrently, biological reconstruction methods, including vascularised bone grafts and tissue engineering approaches, have emerged as viable options in selected patient populations. Despite these advancements, surgical decision-making remains predominantly structure-driven, with limited incorporation of tumour biology, imaging-derived functional data, and systemic treatment response, thereby constraining precision in reconstruction planning.

Despite these advances, several critical gaps persist in skeletal tumour management [2]. There remains a significant disconnect between rapidly evolving knowledge of metastatic biology and its translation into patient-specific surgical strategies. Traditional prognostic scoring systems rely primarily on clinical and radiographic parameters, often failing to capture molecular heterogeneity and treatment responsiveness [5]. Furthermore, the impact of emerging systemic therapies, including targeted agents and immunotherapies, on surgical timing, complication rates, and long-term reconstruction outcomes remains incompletely understood [9]. There is also limited consensus on integrating multidisciplinary data into surgical planning, particularly in complex cases involving extensive bone loss or compromised host biology. Importantly, the absence of a unified, biologically driven and imaging-integrated decision framework contributes to variability in clinical practice and limits the reproducibility of outcomes. These gaps highlight the need for a more cohesive approach that aligns biological insights with practical decision-making [4].

The metastatic process and reconstructive principles are inherently interrelated and can be integrated to optimise patient selection, surgical outcomes, and functional recovery [10]. Orthopaedic oncology, therefore, requires a shift toward precision-based, patient-specific care pathways that incorporate tumour biology, imaging characteristics, and systemic treatment history. In this context, this review advances beyond descriptive synthesis by proposing an integrative clinical framework that systematically links molecular pathways, radiological phenotype, and surgical strategy selection. This approach moves toward actionable decision-making rather than narrative aggregation and supports the broader transition toward precision medicine, where interventions are tailored to the individual biological and clinical phenotype of each patient.

Objectives of the review

This review aims to synthesise current advancements in the biological understanding of skeletal tumour metastasis and examine their implications for contemporary surgical reconstruction strategies. In addition, it seeks to critically evaluate how modern imaging modalities and radiomics-derived biomarkers (quantitative imaging features derived from advanced image analysis) can be comparatively and functionally integrated within surgical planning frameworks, emphasising their role in improving prognostic accuracy and guiding intervention strategies. It further seeks to highlight emerging concepts that support biologically informed, patient-specific decision-making in orthopaedic oncology. Through a structured and analytical synthesis, this review introduces a clinically applicable integrative framework that connects tumour biology, imaging-derived biomarkers, and reconstructive decision-making, thereby addressing a key gap in current literature and enhancing translational and clinical utility.

Methodology

This review was conducted using a structured narrative methodology to ensure methodological transparency while retaining the flexibility required for integrative analysis. A comprehensive literature search was performed across PubMed, Scopus, and Web of Science databases, covering publications from January 2015 to March 2025. The search strategy incorporated combinations of keywords including "skeletal tumours", "bone metastasis", "orthopaedic oncology", "tumour microenvironment", "radiomics", "functional imaging", "endoprosthetic reconstruction", and "limb salvage surgery", with additional use of controlled vocabulary (MeSH terms) and Boolean operators (AND, OR) to refine search sensitivity and specificity. A representative PubMed search strategy included: ("skeletal tumours" OR "bone metastasis") AND ("MRI" OR "PET/CT" OR "radiomics") AND ("orthopaedic oncology" OR "surgical planning"), demonstrating the application of Boolean logic to optimise search retrieval.

The study selection process was conducted in a stepwise manner, involving initial screening of titles and abstracts followed by full-text review of potentially eligible articles. Duplicates were removed prior to screening. The screening workflow followed a structured sequence of identification, screening, eligibility assessment, and final inclusion, consistent with Preferred Reporting Items for Systematic reviews and Meta-Analyses (PRISMA)-informed principles. Articles were assessed for relevance, methodological quality, and clinical applicability. Formal risk-of-bias assessment tools were not applied due to the heterogeneity of included study designs, which is consistent with accepted narrative review methodology; however, a qualitative appraisal framework was used to assess study validity and relevance, considering study design, sample size, consistency of findings, and translational applicability to orthopaedic oncology practice. Although formal risk-of-bias tools such as the Cochrane Risk of Bias tool or Newcastle-Ottawa Scale were not applied, this structured qualitative appraisal provided a systematic approach to evaluating study quality. This approach was selected in preference to formal quantitative bias assessment tools to preserve inclusivity of diverse study designs while maintaining methodological transparency, although it inherently limits comparability and standardised bias quantification. To minimise selection bias, predefined inclusion criteria were applied consistently across all stages of screening, and efforts were made to ensure balanced representation of clinical, imaging, and translational studies.

Studies were included if they addressed one or more of the following domains: (1) molecular mechanisms of skeletal metastasis, (2) advances in diagnostic imaging and radiomics, (3) systemic therapies influencing bone tumour biology, and (4) surgical or reconstructive strategies in orthopaedic oncology. Priority was given to systematic reviews, meta-analyses, prospective clinical studies, and high-impact translational research with clear clinical relevance. Case reports and studies lacking direct applicability to surgical decision-making were selectively excluded unless they provided unique mechanistic insights. Approximately 152 studies were screened, of which 44 met the inclusion criteria and were incorporated into the final synthesis, ensuring adequate representation across all thematic domains.

Data extraction focused on identifying key themes linking tumour biology, imaging characteristics, and surgical outcomes. Rather than performing quantitative synthesis, an analytical framework was applied to integrate findings across disciplines, with explicit emphasis on identifying clinically actionable relationships between imaging biomarkers, biological pathways, and surgical strategies. This approach represents a structured narrative synthesis, integrating findings through thematic and comparative analysis rather than statistical pooling. Comparative evaluation across included studies was performed by identifying recurring patterns, consistencies, and divergences in reported findings, enabling semi-quantitative interpretation of trends without formal statistical pooling. This thematic synthesis was organised into interconnected domains, enabling comparative evaluation of imaging modalities, correlation of molecular mechanisms with radiological phenotypes, and alignment of these factors with surgical decision-making and reconstructive approaches.

Structured tables were utilised to synthesise key findings across studies, facilitating semi-quantitative comparison of imaging modalities, biological mechanisms, and surgical outcomes. While this integrative approach enhances cross-disciplinary synthesis and clinical interpretability, it does not allow statistical effect estimation or direct comparison of outcome measures across studies, representing a recognised limitation relative to systematic review methodologies. Although a formal systematic review protocol was not employed, this structured narrative approach was intentionally selected to enable integrative, cross-disciplinary synthesis of heterogeneous evidence, which would not be feasible using conventional meta-analytic methods while maintaining methodological transparency and clinical relevance. The study selection process followed a structured screening approach consistent with PRISMA-informed principles, although a formal PRISMA flow diagram was not generated due to the narrative design.

No quantitative or statistical synthesis (e.g., meta-analysis or pooled effect estimation) was performed due to heterogeneity in study design, outcomes, and reporting; this limits the ability to derive pooled estimates or perform comparative statistical inference, and findings should therefore be interpreted as qualitative and hypothesis-generating rather than confirmatory.

## Review

Metastatic biology of skeletal tumours

Mechanisms of Bone Tropism and Tumour Homing

Circulating tumour cells exhibit highly regulated mechanisms governing their localisation to bone tissue, a process defined as skeletal metastasis [11]. The seed-and-soil paradigm explains bone tropism by highlighting the selective affinity of tumour cells for the bone microenvironment, which is rich in growth factors, extracellular matrix, and vascular niches [12]. The role of chemokine signalling in this process is prominent, as migration of tumour cells toward bone marrow stromal regions is directed by the CXCL12-CXCR4 axis [13]. This chemotactic concentration gradient facilitates adhesion of tumour cells to endothelial surfaces, followed by their extravasation into the marrow compartment [5].

Integrin and cadherin adhesion molecules further enhance the anchorage of tumour cells within the bone matrix and promote colonisation [9]. Communication with osteoblasts, stromal cells, and endothelial elements establishes a favourable niche that supports tumour cell survival and dormancy [7]. Dormant tumour cells may persist for prolonged periods and undergo reactivation under specific microenvironmental conditions, including hypoxia and inflammatory signalling. Additional factors, including hypoxia-inducible factors and extracellular vesicles, regulate niche preparation and increase metastatic efficiency [14].

Among these mechanisms, the CXCL12-CXCR4 axis and adhesion-mediated colonisation represent key early steps in metastatic establishment, with direct implications for imaging detection and therapeutic targeting. These pathways influence tumour distribution patterns within bone, which may correlate with imaging phenotypes such as marrow infiltration patterns on MRI and metabolic activity on PET/CT. However, integration of such biological insights into radiological interpretation and surgical decision-making remains limited. These coordinated processes represent early and potentially targetable stages of metastatic colonisation within skeletal tissue.

Molecular Pathways Driving Bone Destruction and Formation

After colonisation, tumour cells actively impair bone homeostasis through complex molecular signalling pathways that regulate osteoclast and osteoblast activity [15]. The RANK/RANKL/OPG axis is central to osteoclastogenesis, and increased expression of RANKL promotes differentiation and activation of osteoclast precursors, resulting in enhanced bone resorption [8]. This pathway is further amplified by tumour-derived factors, leading to osteolytic lesions characterised by structural weakening and increased susceptibility to fractures [2]. Simultaneously, certain malignancies stimulate osteoblastic activity through the secretion of endothelin-1 and bone morphogenetic proteins, resulting in unregulated and disorganised bone formation [16]. Cytokines such as transforming growth factor-β and interleukin-6 mediate a bidirectional process, whereby degradation of bone matrix releases growth factors that further promote tumour growth [17]. This establishes a sustained tumour-bone feedback loop that drives disease progression and represents a critical therapeutic target.

The development of molecular understanding indicates that exosomal signalling, immune modulation, and metabolic adaptation are involved in the formation of the metastatic niche [6]. Tumour-derived exosomes contain regulatory molecules that enhance osteoclastic activity and inhibit immune surveillance [3]. Changes in immune cell populations, including macrophage polarisation and T-cell dysfunction, contribute to tumour persistence in bone [12]. Metabolic reprogramming facilitates tumour survival in hypoxic and nutrient-variable marrow environments [17]. These interconnected pathways underscore the biological complexity of skeletal metastasis and provide a mechanistic basis for integrating molecular insights with imaging and surgical decision-making. Table 1 shows the principal molecular mechanisms and their biological implications in skeletal metastasis.

**Table 1:** Key molecular mechanisms and biological implications in skeletal metastasis CXCL12 - C-X-C motif chemokine ligand 12; CXCR4 - C-X-C chemokine receptor type 4; RANK - receptor activator of nuclear factor kappa-B; RANKL - receptor activator of nuclear factor kappa-B ligand; BMPs - bone morphogenetic proteins; TGF-β - transforming growth factor beta; IL-6 - interleukin-6

Mechanism	Key mediators	Cellular targets	Biological effect	Clinical implication	Surgical/clinical relevance priority	References
Chemotactic homing	CXCL12, CXCR4	Tumour cells, stromal cells	Directed migration to bone niches	Early metastatic localisation	Moderate – informs early detection patterns and imaging surveillance strategies	Khan et al. [14]
Cell adhesion and colonization	Integrins, cadherins	Endothelium, bone matrix	Stable anchorage and niche establishment	Persistence of tumour cells	Moderate – contributes to lesion stability and potential resistance to therapy	Zhang [3]
Osteoclast activation	RANK, RANKL	Osteoclast precursors	Increased bone resorption	Osteolytic lesions, fracture risk	High – directly influences fracture risk assessment and need for prophylactic fixation	Trovarelli et al. [8]
Osteoblastic stimulation	Endothelin-1, BMPs	Osteoblasts	Excess bone formation	Osteosclerotic lesions	Moderate – impacts bone density and implant fixation strategy	De Leon-Oliva et al. [16]
Tumour-bone feedback loop	TGF-β, IL-6	Tumour and bone cells	Sustained tumor proliferation	Disease progression	High – drives disease progression and influences timing and aggressiveness of surgical intervention	Freeman et al. [17]

Advances in diagnostic and imaging modalities

Diagnostic assessment of skeletal tumours has significantly advanced through the integration of functional and molecular imaging methodologies, extending beyond conventional anatomical evaluation [18].

Role of MRI in Local Disease Assessment

Magnetic resonance imaging (MRI) provides high-resolution visualisation of marrow involvement, soft tissue extension, and neurovascular relationships, enabling precise delineation of tumour margins [12]. Diffusion-weighted imaging and dynamic contrast-enhanced imaging are advanced MRI techniques that provide insights into cellular density and vascular perfusion, thereby contributing to the assessment of tumour aggressiveness [10]. These advanced MRI techniques allow more detailed characterisation of tumour biology beyond structural imaging alone.

MRI demonstrates high sensitivity for the detection of marrow infiltration and soft tissue extension, particularly in early-stage disease. However, its specificity may be limited, as tumour infiltration can sometimes be difficult to distinguish from reactive or inflammatory changes.

Role of PET/CT in Systemic Disease Evaluation

Computed tomography, particularly when combined with positron emission tomography, serves as a critical modality for identifying metabolically active lesions and evaluating systemic disease burden [19]. The use of radiotracers such as fluorodeoxyglucose enables the detection of hypermetabolic tumour sites, facilitating early identification of skeletal metastases and assessment of treatment response. This enables whole-body evaluation of disease distribution, which is essential for staging and treatment planning.

Hybrid imaging modalities integrate structural and metabolic information, thereby enhancing diagnostic accuracy and staging precision [4]. PET/CT demonstrates higher specificity for metabolically active disease and superior whole-body staging capability. However, sensitivity may vary depending on tumour type, lesion size, and radiotracer uptake characteristics.

Comparative Perspective

A comparative overview of imaging performance is summarised in Table 2, highlighting the complementary roles of MRI for local tumour assessment and PET/CT for systemic disease evaluation.

**Table 2:** Comparative evaluation of imaging modalities in skeletal tumour assessment and surgical planning

Modality	Strength	Limitation	Sensitivity/specificity (general)	Clinical role	Surgical relevance
MRI	Excellent soft tissue detail	Lower specificity	High sensitivity	Local staging	Margin planning
CT	Cortical detail	Limited soft tissue contrast	Moderate	Structural assessment	Implant planning
PET/CT	Metabolic activity	Cost, variability	High specificity	Systemic staging	Surgical intent decision
DWI MRI	Early detection	Artefact variability	High sensitivity	Aggressiveness assessment	Risk stratification
Radiomics	Quantitative analysis	Poor standardisation	Variable	Prognosis prediction	Future surgical planning

Comparative Integration of Imaging Modalities

A comparative perspective indicates that MRI is superior for local tumour characterisation and assessment of soft tissue involvement, whereas PET/CT provides essential information on metabolic activity and whole-body disease distribution. Despite these complementary roles, their combined application remains underutilised in standardised surgical planning pathways.

Variability in imaging protocols, acquisition parameters, and interpretation criteria further limits reproducibility and cross-study comparability, particularly in multicentre settings. This variability reduces consistency in how imaging findings are translated into clinical decision-making.

Radiomics and Artificial Intelligence in Imaging

Quantitative imaging through radiomics and artificial intelligence-based analysis has further advanced imaging interpretation by enabling the extraction of high-dimensional data correlated with tumour phenotype and biological behaviour [9]. These approaches allow non-invasive evaluation of tumour heterogeneity and have potential utility in predicting treatment response and disease progression [20].

However, despite their potential, radiomics and AI-based approaches face several limitations. These include a lack of standardisation in image acquisition, variability in feature extraction methods, and limited external validation. Reproducibility across institutions remains a major challenge, restricting their routine clinical adoption and limiting integration into surgical workflows.

Translational Gap in Surgical Application

Despite these advancements, translation of imaging-derived biomarkers into actionable surgical decision-making remains limited, with insufficient integration into risk stratification models, implant selection, and operative planning. Although imaging provides detailed anatomical and functional information, there is a lack of structured frameworks linking specific imaging findings to defined surgical strategies. As a result, radiological data remain underutilised in operative planning.

**Figure 1:**
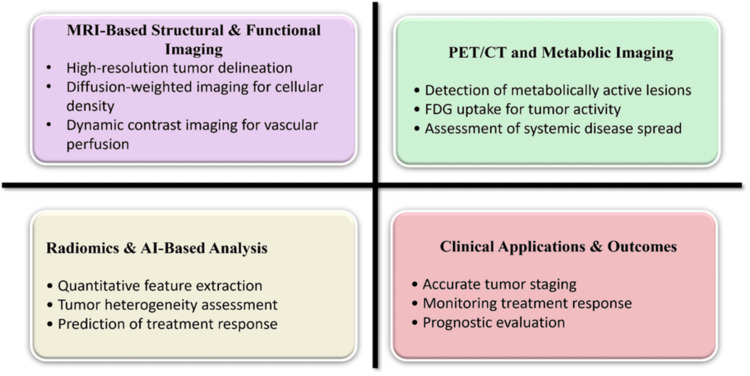
Multimodal imaging and AI integration in tumour evaluation Created by authors using MS PowerPoint (Microsoft, Redmond, WA) FDG - fluorodeoxyglucose

Functional imaging modalities have contributed to bridging the gap between radiological findings and underlying tumour biology. However, their full potential in guiding personalised surgical strategies has not yet been systematically realised. Figure 1 shows the combined roles of MRI, PET/CT, and AI in tumour evaluation and outcomes.

Role of Imaging in Surgical Planning and Prognostication

**Figure 2:**
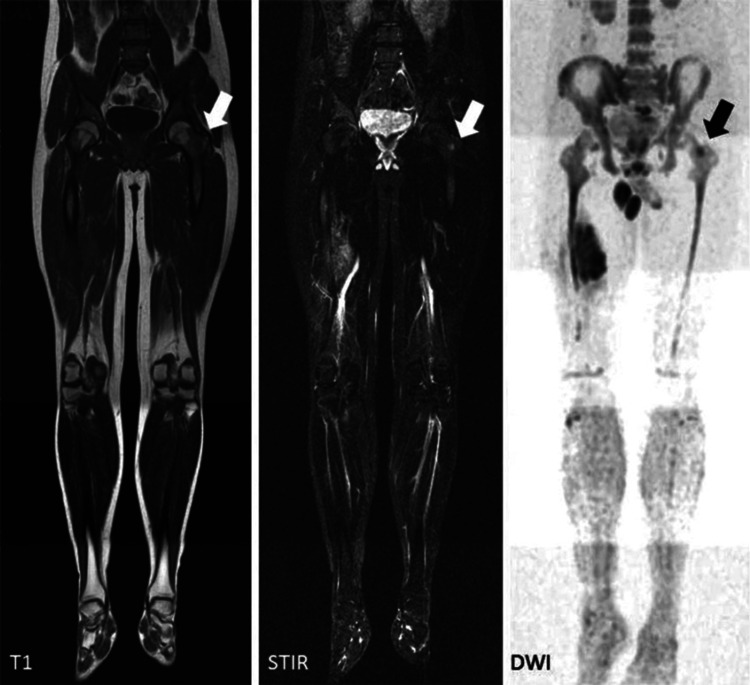
Multimodal MRI evaluation of skeletal tumour demonstrating local extent and marrow involvement Source: Heindel et al. [23] Whole-body MRI sequences, including T1-weighted, short tau inversion recovery (STIR), and diffusion-weighted imaging (DWI), illustrate a primary lesion in the proximal femur with associated marrow infiltration (arrows). T1-weighted imaging demonstrates a hypointense signal corresponding to tumour infiltration, while STIR and DWI sequences show a hyperintense signal indicating increased cellularity and oedema. The imaging also identifies an additional metastatic focus not clearly appreciable on conventional imaging, highlighting the sensitivity of MRI in detecting early marrow involvement and multifocal disease. These findings are critical for surgical planning, as they inform resection margins, the feasibility of limb-salvage procedures, and the need for extended reconstruction strategies.

Imaging has become central to surgical decision-making, enabling precise evaluation of tumour extent and structural integrity of the affected bone [15]. High-resolution imaging allows detailed assessment of cortical destruction, intramedullary extension, and soft tissue invasion, which are critical determinants of resection margins and feasibility of limb-salvage procedures [21]. Computed tomography (CT) provides superior visualisation of cortical bone architecture and plays a key role in preoperative planning of implant positioning and reconstruction strategies [7]. Fracture risk assessment represents a crucial component of imaging evaluation, particularly in metastatic disease [8]. Prediction of pathological fractures is facilitated by quantitative scoring systems supported by imaging findings, guiding the need for prophylactic stabilisation [22]. Imaging biomarkers further contribute to prognostication by reflecting tumour burden, metabolic activity, and response to systemic therapy [6]. This relationship is illustrated in Figure 2, where MRI sequences demonstrate marrow infiltration and multifocal disease directly influencing resection planning.

Representative clinical imaging further illustrates this integration: MRI typically demonstrates marrow infiltration patterns, soft tissue extension, and neurovascular involvement, while PET/CT highlights areas of increased metabolic activity (e.g., elevated standardised uptake value (SUV)), indicating tumour aggressiveness and systemic disease burden. For instance, MRI-defined extensive intramedullary spread with cortical breach may necessitate wider resection margins, whereas PET/CT evidence of multifocal high-SUV lesions may shift management toward palliative stabilisation rather than aggressive limb-salvage surgery. Inclusion of such real diagnostic imaging correlations strengthens the direct linkage between radiological phenotype and operative planning.

Specific imaging findings can directly influence operative strategy; for example, extensive cortical destruction and high Mirel's score support prophylactic fixation, while MRI-defined neurovascular involvement may preclude limb-salvage surgery and necessitate more radical procedures. Similarly, PET/CT-derived high metabolic tumour burden may favour palliative stabilisation over aggressive resection in patients with limited expected survival. Despite these associations, such imaging parameters are not consistently incorporated into standardised surgical decision-making frameworks.

**Figure 3:**
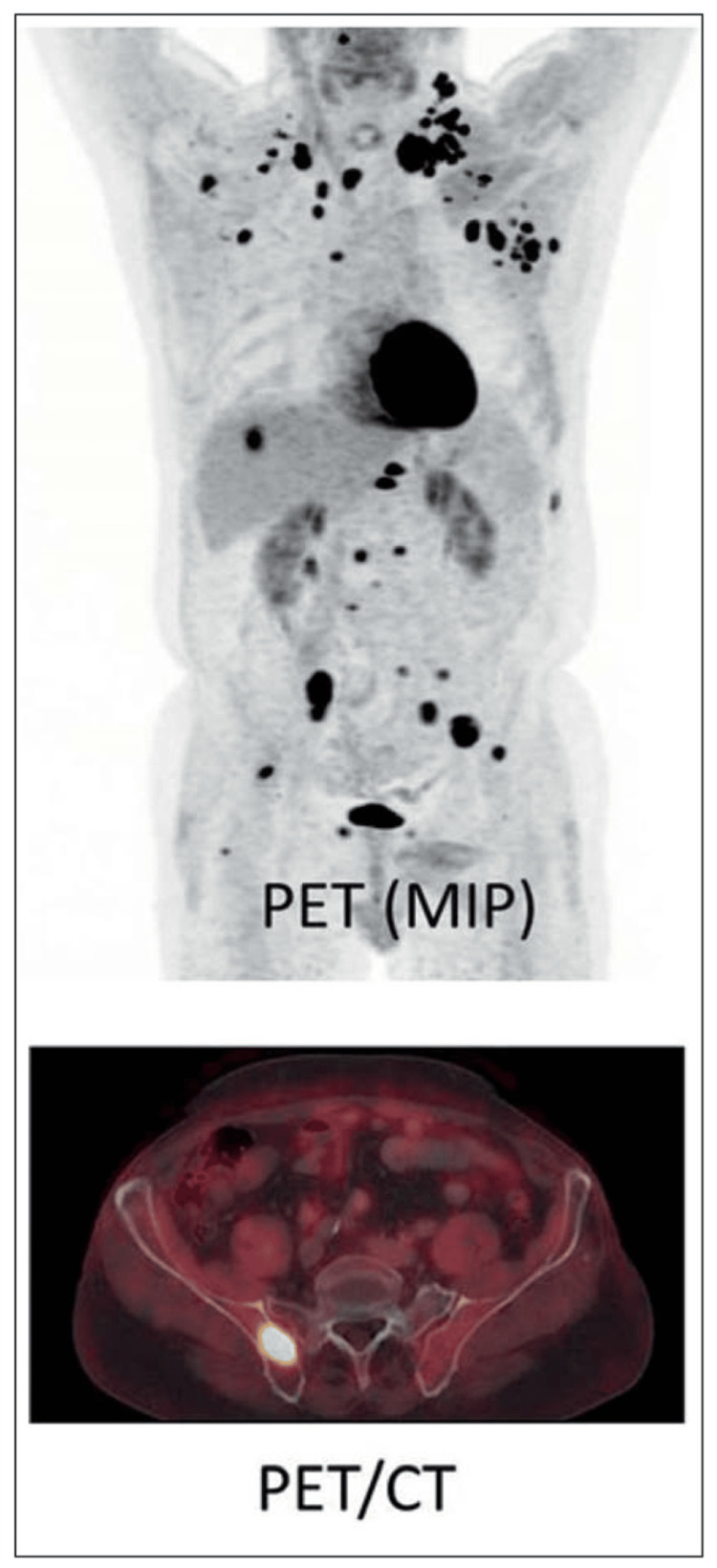
PET and PET/CT imaging demonstrating metabolic tumour burden and systemic disease distribution in skeletal metastasis Source: Heindel et al. [23] Maximum intensity projection (MIP) PET imaging reveals multiple areas of increased radiotracer uptake distributed throughout the axial and appendicular skeleton, indicating widespread metabolically active metastatic disease. The corresponding fused PET/CT image localises areas of high metabolic activity to specific anatomical structures, confirming skeletal involvement and enabling precise lesion localisation. PET/CT demonstrates superior sensitivity in identifying metabolically active lesions that may be inconspicuous on conventional structural imaging. These findings are critical for surgical decision-making, as high systemic tumour burden and multifocal skeletal involvement typically favour palliative stabilisation strategies over aggressive limb-salvage or reconstructive procedures.

From a comparative perspective, MRI provides a detailed assessment of soft tissue involvement and marrow infiltration, whereas CT offers structural precision for reconstructive planning, and PET/CT contributes functional and metabolic insights; however, integration of these complementary modalities into unified surgical algorithms remains limited. This distinction between local tumour extent and systemic disease burden is illustrated in Figure 3, where MRI defines resection parameters while PET/CT demonstrates metastatic distribution influencing surgical intent.

Variability in imaging acquisition protocols, interpretation criteria, and lack of consensus guidelines further restrict consistent application of imaging findings in surgical planning. Serial imaging enables monitoring of treatment response and disease progression, thereby supporting timely adaptation of therapeutic strategies [11]. Changes in lesion size, metabolic activity, and structural integrity serve as important indicators of therapeutic efficacy [23].

To enhance clinical applicability, imaging findings should be incorporated into a structured decision pathway: (1) assessment of structural integrity (CT-based cortical destruction, Mirel's score), (2) evaluation of local tumour extent (MRI-defined marrow and soft tissue involvement), and (3) determination of systemic disease burden (PET/CT metabolic activity and lesion distribution). This stepwise integration enables stratification into surgical pathways, including prophylactic fixation, limb-salvage resection with reconstruction, or palliative stabilisation, thereby translating imaging-derived data into actionable operative strategies.

Although longitudinal imaging provides dynamic assessment of disease evolution, its translation into adaptive surgical planning remains inconsistent, with limited use of serial imaging data in modifying operative timing or reconstruction strategy. Integration of longitudinal imaging data with clinical and molecular parameters enhances predictive accuracy and supports dynamic, patient-specific surgical planning. A comparative overview of imaging modalities for surgical decision making is provided in Table 3.

**Table 3:** Key imaging findings and their implications for surgical decision-making in skeletal tumours SUV - standardised uptake value

Imaging finding	Modality	Interpretation	Surgical decision impact
Cortical destruction >50%	CT	Structural instability	Prophylactic fixation
Marrow infiltration length	MRI	Tumour extent	Resection margin planning
Neurovascular involvement	MRI	Advanced local disease	Limb salvage vs amputation
High SUV uptake	PET/CT	High tumour burden	Palliative vs aggressive surgery
Multifocal lesions	PET/CT	Disseminated disease	Avoid extensive reconstruction
Soft tissue extension	MRI	Local aggressiveness	Wider resection margins

Systemic therapies and their surgical implications

Targeted Therapies and Immunotherapy in Bone Metastases

Systemic therapies have significantly transformed the management of skeletal tumours by altering interactions between tumour biology and the bone microenvironment [24]. Agents such as bisphosphonates and denosumab directly inhibit osteoclast-mediated bone resorption, reducing skeletal-related events including pathological fractures and spinal instability [14]. Bisphosphonates induce osteoclast apoptosis and suppress bone turnover, resulting in stabilisation of osteolytic lesions [7]. Denosumab, a monoclonal antibody targeting RANKL, inhibits osteoclast differentiation and activity, providing effective control of bone destruction [9].

Targeted therapies directed against specific oncogenic pathways have further improved management of bone metastatic disease [25]. Agents targeting vascular endothelial growth factor, epidermal growth factor receptor, and related signalling cascades contribute to reduced tumour proliferation and vascularity in skeletal sites [2]. Immunotherapy has introduced an additional therapeutic dimension by enhancing the host immune response against tumour cells [26]. Immune checkpoint inhibitors, including programmed cell death protein-1 and cytotoxic T-lymphocyte-associated antigen-4 inhibitors, increase T-cell activity and can induce tumour regression in selected malignancies with skeletal involvement [20].

These systemic interventions not only reduce tumour burden but also modify the biological and vascular characteristics of lesions, thereby directly influencing surgical planning, including resection margins, implant selection, and expected healing capacity [24,26]. Despite this, integration of treatment response metrics into surgical decision algorithms remains inconsistent, representing a critical gap in multidisciplinary management. Systemic treatment response should therefore be incorporated into surgical decision-making to optimise outcomes and minimise perioperative complications.

Neoadjuvant and Adjuvant Treatments Influencing Reconstruction Timing

The timing and feasibility of surgical reconstruction in skeletal tumours are significantly influenced by neoadjuvant and adjuvant therapies [27]. Preoperative systemic treatment can induce tumour shrinkage, facilitating limb-salvage procedures and increasing the likelihood of achieving adequate oncological margins. Reduction in tumour volume may simplify surgical procedures and minimise the extent of resection required, thereby preserving surrounding structures and function [19].

Systemic therapies also affect tissue biology, including vascularity, immune response, and regenerative capacity. Antiangiogenic agents may impair wound healing by reducing microvascular density, increasing the risk of postoperative complications [28]. Immunomodulatory therapies alter inflammatory responses, potentially influencing susceptibility to infection and integration of reconstructive implants. These therapy-induced biological changes necessitate careful coordination of treatment sequencing to balance oncologic control with optimal surgical conditions [22].

Adjuvant therapies administered postoperatively contribute to local and systemic disease control, reduce recurrence risk, and improve survival outcomes [14]. However, they may also influence osseointegration, implant stability, and long-term durability of reconstructive constructs, requiring consideration during surgical planning [29]. The timing of adjuvant therapy must be carefully aligned with wound healing and complication risk to ensure safe and effective oncologic management. Table 4 shows major systemic therapies, their mechanisms, biological effects, and implications for surgical reconstruction and clinical outcomes.

**Table 4:** Systemic therapies and reconstruction outcomes in skeletal tumours RANKL - receptor activator of nuclear factor kappa-B ligand

Therapy type	Mechanism of action	Biological effect	Surgical consideration	Clinical outcome impact	Limitations/ trade-offs	References
Bisphosphonates	Osteoclast apoptosis	Reduced bone resorption	Improved bone stability	Decreased fracture risk	Limited effect on tumour burden; long-term use associated with osteonecrosis risk	Khan et al.[14]
Denosumab	RANKL inhibition	Suppressed osteoclast activity	Enhanced control of osteolysis	Reduced skeletal-related events	Potential rebound bone loss after discontinuation; cost considerations	Yang et al. [9]
Targeted therapies	Inhibition of oncogenic pathways	Reduced tumour proliferation	Altered tumour vascularity	Improved disease control	Variable response depending on tumour genetics; resistance may develop	Kroeze et al [25]
Immune checkpoint inhibitors	T-cell activation	Enhanced immune-mediated tumour killing	Modulated inflammatory response	Potential tumour regression	Unpredictable response; immune-related adverse effects impacting perioperative care	Pallumeera et al. [20]
Antiangiogenic agents	Inhibition of vascular growth factors	Reduced vascular supply	Impaired wound healing risk	Delayed recovery potential	Increased risk of postoperative complications and delayed tissue repair	Coleman et al. [28]

Principles of surgical management in skeletal tumours

Indications for Surgery: Curative vs Palliative Intent

Surgical management of skeletal tumours is guided by a comprehensive evaluation of disease extent, patient prognosis, and functional status, with treatment intent broadly classified as curative or palliative [30]. Curative surgery is primarily indicated in selected primary bone malignancies and in cases of solitary metastatic lesions amenable to complete resection with acceptable morbidity [13]. Staging systems, such as the Enneking system and the American Joint Committee on Cancer classification, facilitate assessment of tumour grade, local extent, and metastatic spread, thereby informing surgical planning [31]. Prognostic scoring models play a critical role in determining surgical candidacy in metastatic bone disease [22]. The Mirel's scoring system evaluates fracture risk based on lesion size, location, radiographic characteristics, and pain severity, guiding decisions regarding prophylactic stabilisation [7]. Other prognostic models incorporate variables such as primary tumour type, systemic disease burden, and patient performance status to estimate survival and determine the appropriate extent of surgical intervention [16].

Palliative surgery focuses on symptom relief, preservation of mobility, and improvement of quality of life in patients with advanced disease [6]. Common indications include stabilisation of pathological fractures, decompression of neural structures, and management of refractory pain [13]. In this context, surgical strategies prioritise durability, rapid recovery, and minimal perioperative morbidity [32]. Alignment of surgical goals with patient-centred outcomes remains essential to optimise clinical benefit and functional preservation.

Oncologic Resection Techniques and Margins

Skeletal tumour resection is based on achieving local tumour control through adequate surgical margins [33]. Surgical margins are classified as intralesional, marginal, wide, and radical, reflecting the extent of tumour and surrounding tissue removal. Wide resection, involving excision of the tumour with a margin of normal tissue, is considered the standard approach for most malignant bone tumours to minimise local recurrence [34]. Limb-salvage surgery has become increasingly prevalent, supported by advances in imaging, surgical techniques, and reconstructive options [17]. Successful limb preservation requires meticulous preoperative planning and precise intraoperative execution to ensure oncologic safety while maintaining structural and functional integrity. In contrast, amputation remains indicated in cases of extensive neurovascular involvement, uncontrolled infection, or inadequate soft tissue coverage [14].

En bloc resection is a critical technique for achieving oncologic clearance, involving the removal of the tumour as a single intact specimen without breaching its capsule [26]. This approach reduces the risk of tumour seeding and local recurrence, particularly in high-grade malignancies. Intraoperative imaging guidance and navigation technologies enhance accuracy in defining resection planes and preserving vital anatomical structures [5]. The choice of resection strategy is influenced by tumour biology, anatomical location, and anticipated functional outcomes, underscoring the need for integration of biological and imaging data into surgical planning. Restoration of stability and function following resection requires close alignment between oncologic principles and reconstructive strategies, contributing to improved long-term outcomes in orthopaedic oncology [35].

Innovations in surgical reconstruction techniques

Endoprosthetic Reconstruction and Modular Implants

Endoprosthetic reconstruction has become a standard approach in the management of skeletal tumours, particularly in limb-salvage procedures involving extensive bone resection [36]. Advances in implant design have significantly improved durability, biomechanical performance, and functional outcomes. Modern modular systems allow intraoperative customisation, enabling precise adaptation to patient-specific anatomical and defect requirements [15]. This modularity facilitates efficient assembly, reduces operative time, and enhances surgical flexibility. Material innovations, including titanium alloys and porous coatings, have improved implant stability and osseointegration [17]. Surface modifications enhance biological fixation and reduce the risk of aseptic loosening, a major determinant of long-term implant survival [37]. Improvements in joint articulation and load distribution have further contributed to increased implant longevity and restoration of biomechanical function [2].

Despite these advantages, endoprosthetic reconstruction is associated with complications including infection, mechanical failure, and aseptic loosening, particularly in patients with prolonged survival, necessitating careful patient selection and long-term surveillance.

Patient-specific prosthesis fabrication using advanced imaging and computer-assisted design enables tailored solutions for complex anatomical regions. Three-dimensional planning and manufacturing allow accurate replication of patient anatomy, improving implant fit and functional outcomes [31]. Emerging strategies, including antimicrobial coatings and infection-resistant surfaces, aim to reduce postoperative complications associated with large implants [9]. Endoprosthetic reconstruction provides immediate structural stability and facilitates early mobilisation, particularly beneficial in patients with limited physiological reserve or metastatic disease [38].

Compared to biological reconstruction techniques, endoprosthetic reconstruction offers superior early functional recovery and immediate load-bearing capacity, but may demonstrate inferior long-term biological integration. In contrast, biological reconstruction may provide durable integration but is associated with longer recovery periods and a higher risk of non-union.

Selection of implant type and fixation technique is influenced by patient age, tumour location, expected survival, and functional demands, ensuring alignment of reconstructive strategy with overall clinical objectives. Therefore, the choice of reconstructive approach should be individualised, integrating patient prognosis, tumour biology, and anticipated functional outcomes, rather than relying solely on anatomical considerations.

Biological Reconstruction and Tissue Engineering Approaches

Biological reconstruction strategies aim to restore living bone tissue and achieve long-term integration within the host environment [39]. Allografts remain widely used for reconstruction of segmental bone defects, providing structural support with potential for biological incorporation. However, complications such as graft resorption, non-union, and infection necessitate careful patient selection and technique optimisation [32]. Vascularised bone grafts represent a more advanced biological option, incorporating an intrinsic blood supply to enhance graft viability and promote healing [40]. These grafts demonstrate improved resistance to infection and superior integration compared to non-vascularised constructs [21]. Microsurgical techniques enable transfer of vascularised fibular or iliac crest grafts, particularly in complex defects and younger patients [25].

Tissue engineering approaches have introduced regenerative strategies combining scaffolds, growth factors, and cellular components to promote bone regeneration [35]. Biodegradable scaffolds provide structural templates for new bone formation, gradually being replaced by native tissue over time. The incorporation of osteoinductive factors, such as bone morphogenetic proteins, enhances cellular differentiation and matrix deposition [38]. Advances in three-dimensional printing have expanded reconstructive capabilities by enabling the fabrication of patient-specific implants and scaffolds [31]. These constructs allow precise geometric matching of defects and can be integrated with biological materials to enhance regenerative potential. Ongoing research in regenerative medicine, including stem cell-based therapies and bioactive compounds, aims to improve long-term functional outcomes and durability [19].

**Figure 4:**
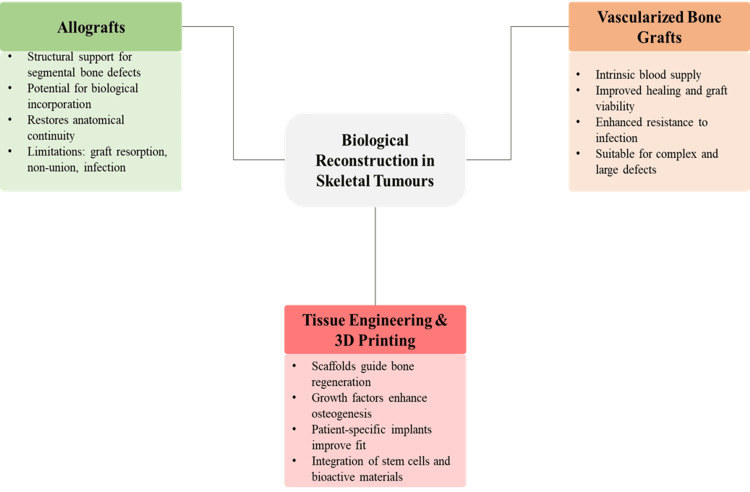
Biological reconstruction strategies in skeletal tumours Created by authors using MS PowerPoint (Microsoft, Redmond, WA)

Biological and engineered reconstruction strategies collectively emphasise restoration of physiological bone function and long-term adaptability, supporting a more individualised and biologically integrated approach to skeletal tumour reconstruction. Figure 4 shows key approaches, including allografts, vascularised bone grafts, and tissue engineering techniques, for restoring bone structure and function.

Multidisciplinary and personalised approaches

Integration of Oncology, Radiology, and Orthopaedic Surgery

Treatment of skeletal tumours requires a multidisciplinary system integrating oncology, radiology, orthopaedic surgery, pathology, and rehabilitation services [31]. Tumour boards facilitate coordinated evaluation of staging, imaging, and treatment strategies, enabling alignment of systemic therapy, imaging interpretation, and surgical planning into a patient-specific pathway [32]. Radiological assessment remains central for defining tumour extent, biological activity, and treatment response, directly influencing surgical feasibility and resection planning [33,34].

Current Limitations in Multidisciplinary Integration

Despite this collaborative structure, multidisciplinary practice often functions in a parallel rather than truly integrative manner, with limited convergence of imaging biomarkers, tumour biology, and systemic therapy response into unified, decision-oriented surgical frameworks [35,36]. This lack of integration results in fragmented decision-making, where key diagnostic inputs are interpreted in isolation rather than collectively.

This fragmentation contributes to variability in clinical practice and underutilisation of advanced diagnostic data, particularly in complex cases requiring precision-based reconstruction strategies [37]. Care pathways improve efficiency, reduce delays, and enhance inter-speciality communication, while perioperative coordination supports improved outcomes and reduced complication rates [38,39].

Need for Structured Decision Frameworks

A key limitation in current practice is the absence of structured models that translate multimodal data into actionable surgical decisions. Effective integration should extend beyond coordination to functional synthesis, where different data streams are actively combined rather than sequentially considered.

In this context, biological pathways (e.g., osteolytic vs osteoblastic activity), imaging phenotypes (e.g., metabolic activity, structural integrity), and systemic treatment response should be collectively mapped to surgical options such as resection extent, implant selection, and timing of intervention.

Proposed Integrated Decision Framework

To operationalise this integration, a structured decision framework can be applied: (1) biological stratification (osteolytic vs osteoblastic phenotype, tumour aggressiveness, molecular drivers), (2) imaging-based assessment (MRI for local extent, CT for structural integrity, PET/CT for metabolic burden and dissemination), (3) systemic therapy response evaluation (tumour reduction, vascularity changes, treatment sensitivity), and (4) surgical mapping (selection between limb-salvage resection, endoprosthetic reconstruction, biological reconstruction, or palliative stabilisation).

This sequential model enables direct translation of multidisciplinary data into reproducible surgical pathways, improving consistency in clinical decision-making.

Role of Radiomics and Predictive Modelling

The incorporation of data-driven tools such as radiomics and predictive modelling offers potential for more objective risk stratification and treatment planning, although standardisation and validation remain limited [40]. Emerging approaches combining radiomic signatures with clinical and molecular data may enable stratification of patients into biologically and radiologically defined subgroups, facilitating more precise and individualised surgical strategies.

A dynamic, feedback-driven multidisciplinary model, incorporating serial imaging and treatment response, can support adaptive decision-making, allowing iterative refinement of surgical planning in response to evolving disease biology.

Challenges and Future Implementation

Furthermore, incorporation of real-world imaging data (e.g., MRI-defined marrow infiltration patterns or PET/CT-derived SUV distribution) into multidisciplinary discussions can enhance concordance between radiological interpretation and surgical execution, reducing variability and improving decision consistency.

However, the absence of standardised protocols and limited prospective validation restricts widespread clinical implementation of such integrated models, highlighting a key area for future research and guideline development.

Such an integrative framework represents a shift from descriptive multidisciplinary care toward a data-driven, decision-oriented model, with potential to enhance reproducibility, optimise functional outcomes, and improve overall clinical effectiveness in orthopaedic oncology (Table 5).

**Table 5:** Multidisciplinary roles in skeletal tumour management

Specialty involved	Primary role	Key contributions	Impact on surgical planning	Clinical outcome benefit	Integration/ decision-making role	References
Oncology	Systemic disease management	Therapy selection, treatment sequencing	Defines the timing of surgery	Improved disease control	Determines surgical feasibility based on tumour response and overall prognosis	Tsukushi et al. [38]
Radiology	Imaging and disease assessment	Tumour staging, response evaluation	Guides resection margins	Enhanced diagnostic accuracy	Provides critical biomarkers (extent, metabolic activity) that influence surgical approach and reconstruction strategy	Gillies et al. [19]
Orthopaedic surgery	Surgical intervention	Resection and reconstruction	Determines operative strategy	Functional restoration	Integrates biological, imaging, and clinical data to execute patient-specific surgical plans	Bläsius et al. [4]
Pathology	Histological evaluation	Tumour grading, margin assessment	Confirms diagnosis and clearance	Reduced recurrence risk	Validates intraoperative margins and informs need for additional resection or adjuvant therapy	Danieli et al. [41]
Rehabilitation services	Functional recovery	Physiotherapy, mobility restoration	Supports postoperative planning	Improved quality of life	Influences reconstruction choice based on expected functional recovery and rehabilitation potential	Louie et al. [32]

Patient-specific decision-making and outcome optimisation

Individual management of skeletal tumours requires integration of clinical, biological, and functional variables to enable personalised treatment strategies [42,43]. Prognostic evaluation incorporates tumour type, metastatic burden, and patient performance status to guide the extent of surgical intervention, while predictive models assist in selecting appropriate reconstructive approaches [40]. Quality of life remains a central consideration, particularly in metastatic disease, with emphasis on functional preservation, pain control, and early mobilisation [34].

However, current decision-making models remain limited by inadequate integration of imaging biomarkers and treatment response parameters, reducing their ability to capture tumour heterogeneity and dynamic disease progression. Incorporation of radiomics and functional imaging has the potential to enhance prognostic precision and provide more objective guidance for surgical planning.

Role of Advanced Technologies in Personalisation

Technological advances such as three-dimensional modelling and computer-assisted surgery enable precise anatomical visualisation and facilitate patient-specific reconstruction [44]. These technologies improve preoperative planning accuracy and allow simulation of resection margins and implant positioning. However, their clinical impact remains constrained by variability in implementation and lack of standardised protocols.

This highlights the need for structured integration of imaging, biological, and computational data into unified clinical workflows.

Structured Patient-Specific Decision Workflow

To enhance clinical applicability, a patient-specific decision workflow can be structured as follows: (1) baseline clinical and prognostic assessment (tumour type, metastatic burden, performance status), (2) biological characterisation (osteolytic vs osteoblastic behaviour, molecular profile), (3) imaging integration (MRI for local extent, CT for structural stability, PET/CT for metabolic burden), (4) treatment response evaluation (changes following systemic therapy, including tumour shrinkage or progression), and (5) surgical strategy selection (curative resection with reconstruction, limb-salvage procedures, or palliative stabilisation).

This structured pathway enables reproducible and transparent decision-making aligned with patient-specific disease characteristics and supports direct translation into clinical practice.

Dynamic and Adaptive Decision-Making

A refined, patient-centred decision framework should incorporate multidimensional inputs, including tumour biology, imaging-derived metrics, systemic therapy response, and functional status, to guide surgical strategy selection in a reproducible manner. A multidisciplinary approach with continuous reassessment supports optimisation of outcomes.

Incorporation of serial imaging and longitudinal treatment response into this framework allows dynamic modification of surgical timing and reconstructive approach, ensuring alignment with evolving tumour behaviour and patient condition.

A dynamic model incorporating serial imaging and longitudinal treatment response enables adaptive surgical planning, aligning intervention strategies with evolving disease characteristics and patient-specific goals, thereby advancing precision-based orthopaedic oncology practice.

Limitations and future recommendations

The inherent limitations of this narrative synthesis include heterogeneity of included studies, variations in study design, and differences in patient populations across skeletal tumour cohorts. The absence of quantitative synthesis limits the ability to establish definitive causal relationships between metastatic biology and surgical outcomes. Furthermore, the lack of statistical pooling precludes formal assessment of heterogeneity, effect size estimation, and cross-study consistency. Potential selection bias, including preferential inclusion of English-language and high-impact studies, may affect representativeness. Variability in reporting of surgical techniques, systemic therapies, and outcome measures further restricts direct comparability. Rapid advancements in oncology and biomaterials may also limit the long-term generalisability of current findings, while the limited availability of long-term follow-up data constrains the assessment of reconstruction durability and functional outcomes.

Although a structured narrative methodology was employed to enhance transparency, this review does not utilise formal systematic review protocols or meta-analytic techniques, which may limit reproducibility and introduce interpretative bias. The integration of imaging, biological, and surgical data is based on currently available evidence but remains constrained by the absence of standardised frameworks and limited prospective validation. Furthermore, variability in imaging protocols and inconsistent reporting of radiomic parameters reduce the reliability of cross-study comparisons and hinder clinical translation. In addition, the absence of formal risk-of-bias assessment tools limits standardised evaluation of study quality, and the reliance on schematic representations rather than real diagnostic imaging restricts direct clinical validation and applicability of proposed concepts. These limitations collectively reduce the ability to derive generalisable conclusions and highlight the need for more structured and quantitative research approaches.

Future multicentre studies integrating molecular profiling, advanced imaging, and surgical outcomes are required to develop biologically informed treatment algorithms. The development of unified prognostic models incorporating imaging, genomic, and clinical variables may improve decision-making accuracy. In particular, incorporation of real-world imaging datasets (MRI and PET/CT) into prospective studies will be essential to validate imaging-derived decision frameworks and enhance translational relevance.

Standardisation of imaging protocols, radiomics methodologies, and reporting frameworks is essential to enable consistent incorporation of imaging biomarkers into surgical workflows. Prospective validation of these tools is necessary to establish their clinical utility and reproducibility. Longitudinal studies are needed to evaluate the impact of emerging systemic therapies on reconstruction durability and complication rates. Further advancements in biomaterials, tissue engineering, and patient-specific implant design are expected to improve long-term stability and functional outcomes.

Future research should also focus on operationalising integrative frameworks into clinically implementable algorithms, including decision-support tools that map tumour biology, imaging findings, and treatment response to specific surgical strategies. Where feasible, the incorporation of semi-quantitative or quantitative methodologies in future studies may enhance analytical rigour and enable more robust comparison across clinical outcomes. Prospective validation of such models in multidisciplinary settings will be critical to establish their reproducibility and clinical utility.

Future research should prioritise the development and validation of integrated, multidisciplinary decision frameworks that systematically combine tumour biology, imaging analytics, and surgical strategy, thereby enhancing precision, reproducibility, and clinical applicability in skeletal tumour management.

## Conclusions

The progress in skeletal tumour management reflects an evolving integration of metastatic biology, advanced imaging, and surgical innovation, enabling more precise and individualised treatment approaches. Improved understanding of tumour-bone interactions supports more accurate prediction of disease behaviour, while modern imaging techniques enhance staging, prognostication, and operative planning. Advances in systemic therapies further influence tumour biology, surgical timing, and postoperative outcomes. Surgical strategies have progressively shifted toward limb-salvage approaches, supported by advancements in endoprosthetic design, biological reconstruction, and multidisciplinary, patient-centred care.

Despite these developments, a critical limitation remains the absence of unified, decision-oriented frameworks that systematically integrate biological insights, imaging-derived biomarkers, and clinical parameters into surgical planning. This review addresses this gap by proposing an integrative conceptual model that links tumour biology, radiological phenotype, and reconstructive strategy, facilitating more consistent and evidence-informed decision-making. Importantly, this framework has been further refined into a clinically structured, stepwise approach that aligns biological characterisation, imaging findings (MRI, CT, PET/CT), and systemic therapy response with specific surgical pathways, thereby improving its practical applicability in multidisciplinary settings. Future progress in orthopaedic oncology will depend on the development and validation of standardised, multidisciplinary frameworks incorporating molecular profiling, advanced imaging analytics, and longitudinal treatment response data. Integration of real-world diagnostic imaging and prospective validation of such decision models will be essential to translate conceptual frameworks into routine clinical practice. Such approaches have the potential to enhance prognostic precision, optimise surgical strategy selection, and improve long-term functional and oncologic outcomes.
